# Genetic Variability and Phylogeny of High Risk HPV Type 16, 18, 31, 33 and 45 *L1* Gene in Greek Women

**DOI:** 10.3390/ijms13010001

**Published:** 2011-12-22

**Authors:** Chara Kleio Ntova, Christine Kottaridi, Aikaterini Chranioti, Aris Spathis, Dimitrios Kassanos, Evangelos Paraskevaidis, Petros Karakitsos

**Affiliations:** 1 Department of Cytopathology, Attikon General University Hospital, Athens University Medical School, Chaidari, 12462, Greece; E-Mails: xntova@gmail.com (C.K.N.); ckottaridi@gmail.com (C.K.); kat_chranioti@yahoo.gr (A.C.); arisspa@gmail.com (A.S.); 2 3rd Department of Obstetrics and Gynaecology, Attikon General University Hospital, Athens University Medical School, Chaidari, 12462, Greece; E-Mail: deptobgyn@attikonhospital.gr; 3 Department of Obstetrics and Gynecology, Faculty of Medicine, University Hospital of Ioannina, Ioannina 45110, Greece; E-Mail: eparaske@cc.uoi.gr

**Keywords:** HPV, high risk, phylogeny, cervical neoplasia, Greece

## Abstract

The present study explores nucleotide variability, phylogeny and association with cervical neoplasia in high risk HPV types 16, 18, 31, 33 and 45 collected from Greek women. Of the 1894 women undergoing routine cervical cytology examination, 160 samples test positive for single infections of HPV type 16 (*n* = 104), HPV 31 (*n* = 40), HPV 33 (*n* = 7), HPV 18 (*n* = 5), and HPV 45 (*n* = 4) were typed by microarrays method, amplified by PCR then sequenced and phylogenetically analyzed. For HPV 16, 9 variants with nucleotide variations were included into the study. For HPV 31, 33, 18 and 45, nucleotide variations were identified in 6, 4, 2 and 3 variants, respectively. The Bayesian inference and Maximum Parsimony methods were used in order to construct the phylogenetic trees. When types were analyzed independently HPV 16 (European and non-European) and HPV 18 (African and non-African) formed distinct clades. The genomic characterization of HPV variants will be important for illuminating the geographical relatedness and biological differences and for the determination of their risk.

## 1. Introduction

Persistent infection of oncogenic HPV types is acknowledged as a major risk factor for the development of cervical carcinoma [1,2]. Most of these high-risk types are phylogenetically clustered with *Human papillomavirus 16* or *Human papillomavirus 18* [3]. According to Bernard *et al*. [4] the classification of the family *Papillomaviridae* has been expanded and contains 29 genera formed by 189 papillomavirus (PV) types isolated from humans (120 types), non-human mammals, birds and reptiles (64, 3 and 2 types respectively). According to de Villiers *et al*. [5] genomic sequence similarities are used for their definitions to types, subtypes and variants. Isolates which differ within the same type by 2 to 10%, compared to prototype, are classified as subtypes, while isolates are classified as variants when they differ from 0 to 2% from the prototype. HPV types 16, 31, 33 belong to species 9 and HPV types 18, 45 belong to species 7 [5]. The ICTV has not set standards for the definition of taxonomic levels lower than species. Chen *et al*. [6,7] define variants of the same type as distinct lineages, when there is an approximate cut off of 1% difference between genomes and differences between genomes in 0.5%–1% range are designated as sublineages.

Analysis of HPV variants diversity worldwide is of great importance as the HPV sequence data bases which are created, are useful for epidemiological and evolutionary studies, for the development of accurate diagnostic tests and for efficient vaccine design. The purpose of the present study was to analyze for the first time in Greece the intratype variability of 160 cervical samples positive for HPV 16, 18, 31, 33 and 45 in order to add information on the circulation of HPV variants in Greece.

## 2. Results

Molecular characterization of HPV 16, 31, 33, 18 and 45 variants within the *L1* (MY9/11) region was undertaken in 160 positive samples with single infections: 104 for HPV 16, 40 for HPV 31, 7 for HPV 33, 5 for HPV 18 and 4 for HPV 45. According to cytological diagnosis, samples were grouped into five categories: within normal limits (WNL) (*n* = 21, 13.1%), Atypical Squamous Cells of Undetermined Significance (ASCUS) (*n* = 12, 7.5%), Low grade lesions (LGSIL) (*n* = 62, 38.75%), High grade lesions (HGSIL) (*n* = 57, 35.6%) and Squamous Cell Carcinoma (SCC, *n* = 7, 4.4%).

**Figure 1:**
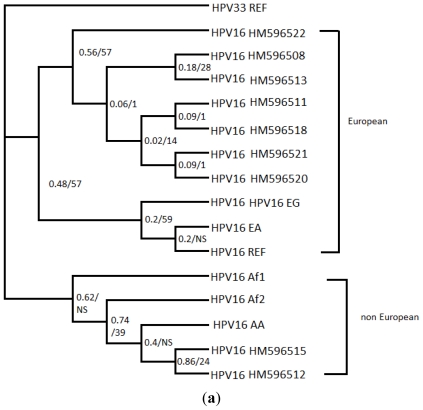

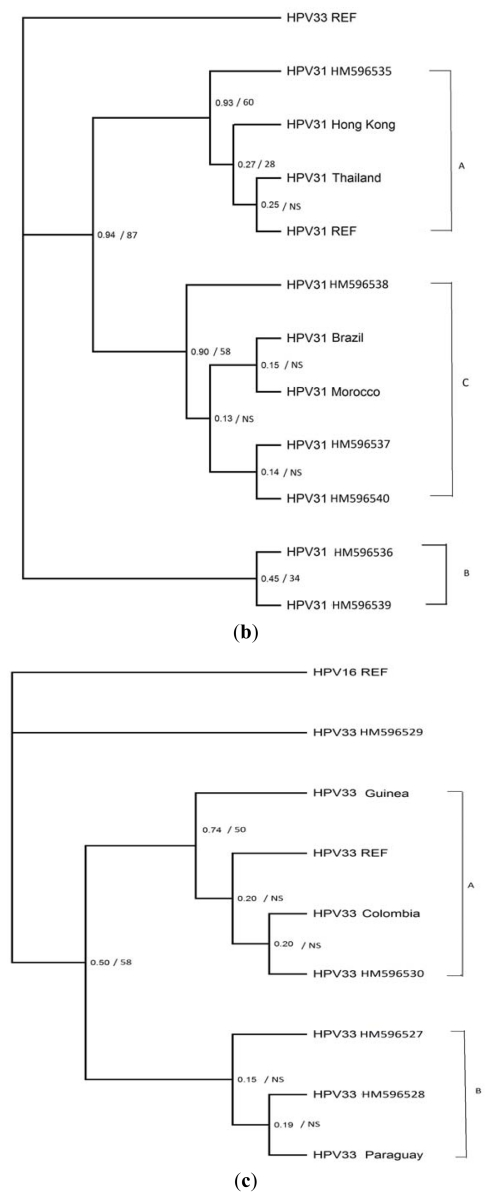

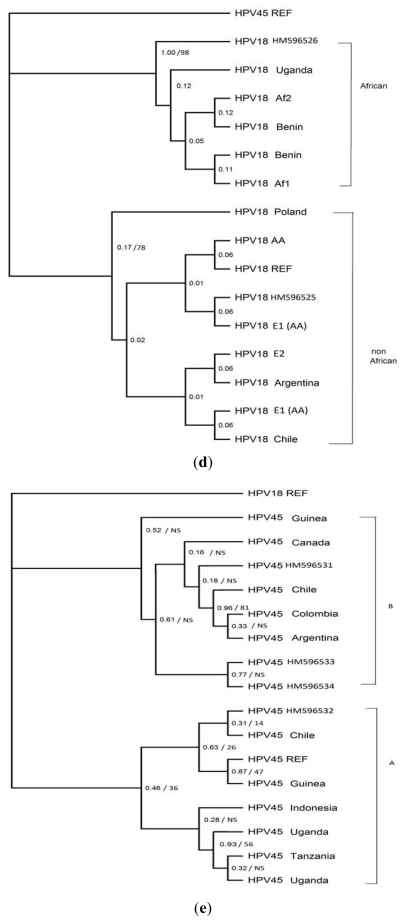
Bayesian inference (BI) tree (50% majority rule consensus tree) based on sequences of *L1* (MY9/11) gene for HPV types. Numbers at nodes indicate posterior probabilities and bootstrap support in the BI and maximum parsimony (MP) analyses, respectively (BI/MP). NS denotes nodes that are not supported. (**a**) Tree of variants of HPV16 found in this study, along with HPV reference sequence and variants of HPV 16 retrieved from GenBank: HPV 16 EG (European German, AF536179), HPV 16 AA (Asian American, AF402678), HPV 16 Af2 (African type 2, AF472509), HPV 16 Af1 (African type 1, AF536180). In the tree of HPV 31 (**b**) some variants of HPV 31 have been submitted previously [10] to GenBank database: HPV 31 Hong Kong (DQ057327), HPV 31 Thailand (DQ057331), HPV 31 Brazil (DQ057330), and HPV 31 Morocco (DQ057332). In the tree of HPV 33 (**c**) variants of HPV 33 have been submitted previously by Stewart *et al*. 1996 [11] to GenBank database: HPV 33 Guinea (U45897), HPV 33 Colombia (U45895), HPV 33 Paraguay (U45896). In the tree of HPV 18 (**d**) variants of HPV 18 have been submitted previously by Stewart *et al*. [11] and Chen *et al*. [6] to GenBank database: HPV 18 Uganda (U45893), HPV 18 Benin (U45894, U45892), HPV 18 Poland (U45891), HPV 18 Argentina (U45889), HPV 18 Chile (U45890), HPV 18 Af2 (African type 2, Qv17199), HPV 18 Af1 (African type 1, Qv21444), HPV 18 E1 AA (European Asian American, Qv03132, Qv16302), HPV 18 E2 (European, Qv21751). In the tree of HPV 45 (**e**) variants of HPV 45 have been submitted previously to GenBank database: HPV 45 Guinea (U45910, U45906), HPV 45 Canada (U45911), HPV 45 Chile (U45908, U45912), HPV 45 Colombia (U45909), HPV 45 Argentina (U45907), HPV 45 Indonesia (U45913), HPV 45 Uganda (U45916, U45915), and HPV 45 Tanzania (U45910).

For HPV 16, 104 samples were analyzed for *L1* gene (MY9/11 region). Of the 104 isolates of HPV 16 sequenced, 9 representative isolates were selected, because they contained at least one unique nucleotide sequence variation in comparison to other isolates of the same type. The majority of variants (93%) were European and among these the sequence of HM596520 was the most frequent (84.6%) (Table 1). From the 384 bp analyzed for *L1* region, 13 base substitutions were detected, 6 of which resulted in amino acid changes and the remaining were silent changes (Table 2a). The isolates of HPV 16 were distributed into previously defined variant lineages of HPV 16 for the same region of *L1* gene, that were retrieved from GenBank database and two big groups were created [7]. Figure 1a shows the Bayesian inference (BI) consensus tree of HPV 16 variants. The best-fit model applied to MrBayes was HKY + I + G, which was selected according to AIC in MrModeltest 2.3 [8] and HPV 33 reference (M12732) sequence was used as the outgroup taxon. In the first group the isolates are clustered with European (EG, reference) and East Asian variants and in the second group are clustered with African (Af1, Af2) and Asian American (AA) variants. Six new nucleotide sequence variations were detected (T6824C, A6914G, T6824G, C6888T, A6994C) in this study (Table 2). The other variants identified to circulate in Greece, have been described before by Yamada *et al*. and Stewart *et al*. [9]. As far as the intratype variability is concerned, the evolutionary divergence between variants of HPV 16 ranged from 0% to 1.9%. The average evolutionary divergence within HPV 16 was 0.7% and the non-European variant (HPV16 HM596515) seems to be the most differentiated (1.9%).

For HPV 31, 40 sequences were sequenced and analyzed for *L1* (MY9/11) gene and 6 variants were found. The most frequent isolate HPV31 HM596540 (Table 1) was clustered with American and African variants and included the 65% of HPV 31 sequences analyzed. Of the 6 variants found, 5 have been described before and 1 (A6647C) is new (Table 2b). None of the sequences analyzed was identical to the prototype (HPV 31 REF, J04353). From the 351 bp analyzed 11 base substitutions were detected, of which only one at nt 6846 resulted in amino acid substitution (Table 2b). Phylogenetic trees for HPV 31 were constructed using HPV 31 variants identified along with sequences from other parts of the world submitted previously to the GenBank database by Calleja-Macias *et al*. and classified to lineages according to Chen *et al*. [7,10]. The best-fit model applied to MrBayes was HKY, which was selected according to AIC in MrModeltest 2.3 [8] and HPV 33 reference sequence was used as the outgroup taxon. In the BI tree created (Figure 1b), variants were classified into two main groups, the first one includes the reference sequence and Asian variants and the second group includes American and African variants. As far as the intratype variability is concerned, the evolutionary divergence between variants of HPV 31 ranged from 0.3% to 1.7%. The average evolutionary divergence within HPV 31 was 1.1% and the isolate HM596536 seems to be the most distant as it differentiates 2% with the reference sequence (HPV 31 REF, J04353).

Among the 7 HPV 33 samples examined, 4 variants were detected. The isolate HM596530 was the most frequent, as it was detected in the 57% of HPV 33 samples analyzed (Table 1). The variant (A6673G) is new and the 3 other variants have been described before (Table 2). Limited variation was found among these variants with only four point nucleotide changes, of which only one (nt 6748) resulted in amino acid substitution. Phylogenetic trees for HPV 33 were constructed using the variants detected along with those from other parts of the world submitted previously by Stewart *et al.*, 1996 and classified to lineages according to Chen *et al*. [7,11]. In the tree produced by the BI analysis (Figure 1c), the isolate HM596530 seems to differentiate more than the other variants and forms a group along with the reference sequence (HPV 33 REF, M12732) and variants from Colombia and Guinea. The best-fit model applied to MrBayes was HKY + G, which was selected according to AIC in MrModeltest 2.3 [8] and HPV 16 reference sequence was used as the outgroup taxon. As far as the intratype variability is concerned, the evolutionary divergence between variants of HPV 33 ranged from 0.3 % to 0.8 %. The average evolutionary divergence within HPV 33 was 0.5%.

For HPV 18, 2 variants were detected from the five different sequences analyzed. The most frequent isolate was the HPV18 HM596525 and 4 from the 5 sequences analyzed, belong to that (Table 1). Limited variation was found between HPV 18 variants. From the 383 bp aligned, eight point nucleotide changes were detected and only one (nt 6748) resulted in amino acid substitution. In the tree produced by the BI analysis for HPV 18, the isolate HM596525 was clustered with the prototype sequence (HPV 18 REF, X05015). Two distinct groups were formed in that tree (Figure 1d). The best-fit model applied to MrBayes was HKY + I, which was selected according to AIC in MrModeltest 2.3 [8] and HPV 45 reference sequence (X74479) was used as the outgroup taxon. The average evolutionary divergence within HPV 18 was 1.6%. The isolate HM596526 appears to differ in comparison to the reference sequence to a percentage of 2.1%.

From the 4 sequences analyzed for HPV 45, 4 variants were detected one of which is new (A6665C) (Table 2). Limited variation was detected and from the 365 bp aligned, nine nucleotide changes were detected and only 3 (nt 6665, nt 6676, nt 6705) resulted in amino acid substitution. In the tree produced by the BI analysis for HPV 45 (Figure 1e) two big groups emerged from the phylogenetic analysis. The variant lineages were named according to Chen *et al*. [6] The best-fit model applied to MrBayes was GTR + G, which was selected according to AIC in MrModeltest 2.3 [8] and HPV 18 reference sequence (X05015) was used as the outgroup taxon. As far as the intratype variability is concerned, the evolutionary divergence between variants of HPV 45 ranged from 0.3% to 1.4%. The average evolutionary divergence within the type HPV 45 was 0.9% and the new variant (A6665C) of HM596534 was the most divergent (2.2%), in comparison to the reference sequence (HPV 45 REF, X74479).

## 3. Experimental Section

### 3.1. Sample Collection

All HPV positive samples already typed with single infections for HPV types 16, 18, 31, 33 and 45 were selected from the HPV DNA bank of the Cytopathology Department. A total of 1894 women undergoing routine cervical cytology examination joined the present study that took place in “Attikon” General University Hospital from April 2009 until December 2010. A total of 943 women were negative for HPV. Of the 951 positive samples 448 were found to have multiple infections either with high or low risk HPV types. Of the remaining 503 with single infections, 183 cervical brush specimens were used in the present study as they had single infections with HPV 16, 18, 31, 33 and 45. Of the 183 samples which were positive for single infection, the 160 were both and PCR amplified and sequenced and included in the present study. A liquid-based cytology (ThinPrep Pap-Test^®^) sample was taken following consent for study participation. The cytologic specimens were originated from different regions all over Greece. In order to explore the intratype phylogeny of the five most oncogenic HPV types, 104 sequences were used for HPV 16, 40 for HPV 31, 7 for HPV 33, 5 for HPV 18 and 4 for HPV 45.

### 3.2. DNA Extraction and HPV Typing

DNA was extracted, amplified and analyzed using microarrays method, by CLART^®^ Human Papillomavirus 2 kit according to the manufacturer’s instructions (GENOMICA, Madrid, Spain). Briefly, 1 mL of homogeneous sample was placed to a sterile 1.5 mL microcentrifuge tube and centrifuged for 10 min at 12,000 rpm in order to be in a pellet form. The supernatant was discarded and the pellet was resuspended with 1 mL of sterile water and centrifuged for 10 min at 12,000 rpm. DNA extraction method was followed by adding 180 μL of lysis buffer T1 and 25 μL of proteinase K into the precipitate and incubating the sample in a thermomixer at 56 °C for 1 h and 550 rpm. At the end of DNA extraction method, 100 μL of eluted DNA recovered and stored at −20 °C.

Five microliters of eluted DNA were used for the PCR amplification. HPV DNA amplification was achieved by using biotinylated primers designed to amplify a 450 bp fragment within the highly conserved *L1* (PGMY9/11) region of the virus. Elimination of false negative results and PCR failure due to several PCR inhibitors present inside the sample, was achieved by using two internal controls: (a) genomic DNA control (a pair of primers that amplify a 892 bp fragment of human *CFTR* gene; and (b) control of the amplification reaction (a pair of primers that amplify a 1202 bp fragment of modified plasmid). Hybridization of the amplified PCR product with immobilized matching type-specific DNA probes took place on a low-density microarray tube (Array Tube System, CLONDIAG Chip Technologies GmbH). The data obtained in each analysis were processed automatically by the system. CLART^®^ HPV 2 kit allowed the detection of infection and co-infection of 35 different HPV genotypes, 20 HR-HPV: 16, 18, 26, 31, 33, 35, 39, 45, 51, 52, 53, 56, 58, 59, 66, 68, 70, 73, 82 & 85 and 15 LR-HPV: 6, 11, 40, 42, 43, 44, 54, 61, 62, 71, 72, 81, 83, 84 & 89.

### 3.3. PCR and Sequencing

Amplification of *a* ≈ 380-bp HPV-specific segment from the *L1* (MY9/11) ORF was performed using the sense primer MY09 (nt 6584) 5′-CGTCCMARRGGAWACTGATC-3′ and the reverse primer MY11 (nt 7035) 5′-GCMCAGGGWCATAAYAATGG-3′. Five microliters of DNA were added to 45 μL of reaction mix containing 1× PCR buffer, 0.2 mM dNTPs, 4 mM MgCl_2_, 0.3 μM of each primer, and 0.025 U/μL of AccuPrime™ Taq DNA Polymerase high Fidelity (Invitrogen). The cycling conditions were as follows: 94 °C for 1 min, 1 cycle; then, 94 °C for 30 s, 50 °C for 30 s, and 72 °C for 30 s, for 35 cycles using the Eppendorf Mastercycler personal thermocycler (Applied Biosystems, Foster City, CA). This region of *L1* gene corresponds in the HPV 16 genome to nucleotides (nt) 6589 to 6969, in the HPV 31 genome to nucleotides (nt) 6509 to 6865, in the HPV 33 genome to nucleotides (nt) 6546 to 6925, in the HPV 18 genome to nucleotides (nt) 6559 to 6944, in the HPV 45 genome to nucleotides (nt) 6577 to 6941. PCR products were subjected to electrophoresis in 1% agarose (Applichem) using 1× TBE buffer (Applichem) and visualized under UV light.

PCR products were purified using commercially available spin columns (Invitrogen PCR purification Kit, Pure Link) and then they were sequenced directly via automated sequencing using both forward and reverse PCR primers. The sequences of *L1* (MY9/11) gene determined for this study have been deposited in GenBank under the following accession numbers: of HPV 16: HM596508-HM596522, of HPV 18: HM596525, HM596526, of HPV 33: HM596527-HM596530, of HPV 45: HM596531-HM596534 and of HPV 31: HM596535-HM596540.

### 3.4. Phylogenetic Analysis

Phylogenetic trees for HPV 16, 31, 33, 18 and 45 isolates were constructed using the variants of *L1* (MY9/11) sequences obtained in this study. The alignment and the correction of sequences of *L1* gene were done by using the Codon Code Aligner program (v.3.5.2.). Analyses for phylogenetic inference were conducted using three methods: Neighbor-Joining (NJ) Maximum Parsimony (MP) and Bayesian Inference (BI). Nucleotides were used as discrete, unordered characters. Neighbor-joining (NJ) trees were constructed using Kimura two-parameter correction methods of MEGA 4 [12]. To assess the confidence of branching patterns of NJ trees, 1000 bootstrap replicates were performed. The MP analysis was performed using PAUP* v.4.0b10 [13], with heuristic searches using stepwise addition of sequences. Gaps were treated as missing characters. Confidence in the nodes was evaluated by 1000 bootstrap replicates [14]. The Bayesian analysis (BI) was performed with MrBayes ver. 3.1 [15] applying in the dataset the parameters of the substitution model suggested by MrModeltest 2.3 [8] according to the Akaike information criterion (AIC) [16]. Support for nodes was assessed with the posterior probabilities of reconstructed clades as estimated by MrBayes 3.1 [15]. In order to make the sequence analysis and find the different variants out of the 160 sequences the program Dambe [17] was used. Representative isolates were selected, because they contained at least one unique nucleotide sequence variation in comparison to other isolates of the same type. Trees were visualized using TreeView [18]. All reference sequences were taken from the Los Alamos-based HPV sequence database [19]. In the tree of HPV 16 (Figure 1a) apart from the variants found in this study we have used variants of HPV 16 retrieved from GenBank: HPV 16 EG (European German, AF536179), HPV 16 AA (Asian American, AF402678), HPV 16 Af2 (African type 2, AF472509), HPV 16 Af1 (African type 1, AF536180). In the tree of HPV31 (Figure 1b) some variants of HPV 31 have been submitted previously [10] to GenBank database: HPV 31 Hong Kong (DQ057327), HPV 31 Thailand (DQ057331), HPV 31 Brazil (DQ057330), and HPV 31 Morocco (DQ057332). In the tree of HPV 33 (Figure 1c) variants of HPV 33 have been submitted previously by Stewart *et al*. 1996 [11] to GenBank database: HPV 33 Guinea (U45897), HPV 33 Colombia (U45895), and HPV 33 Paraguay (U45896). In the tree of HPV 18 (Figure 1d) variants of HPV 18 have been submitted previously by Stewart *et al*. [11] and Chen *et al*. [6] to GenBank database: HPV 18 Uganda (U45893), HPV 18 Benin (U45894, U45892), HPV 18 Poland (U45891), HPV 18 Argentina (U45889), HPV 18 Chile (U45890), HPV 18 Af2 (African type2, Qv17199), HPV 18 Af1 (African type1, Qv21444), HPV 18 E1 AA (European Asian American, Qv03132, Qv16302), HPV 18 E2 (European, Qv21751). In the tree of HPV 45 (Figure 1e) variants of HPV 45 have been submitted previously to GenBank database: HPV 45 Guinea (U45910, U45906), HPV 45 Canada (U45911), HPV 45 Chile (U45908, U45912), HPV 45 Colombia (U45909), HPV 45 Argentina (U45907), HPV 45 Indonesia (U45913), HPV 45 Uganda (U45916, U45915), and HPV 45 Tanzania (U45910).

## 4. Discussion

In the present study, molecular characterization of HPV 16, 18, 31, 33 and 45 variants within the MY09/11 *L1* genomic region was performed in 160 positive samples with different cytological diagnosis. The viral persistence and the development of high grade lesions associated with specific HPV variants has been the objective of many studies worldwide as genome variations may be relevant to virus infectivity and pathogenicity [20–23]. This association may be explained by existence of specific viral variant epitopes in L1 capsid which are targets for neutralizing antibodies [24–28].

HPV 16 prototype (K02718) was isolated from a German cervical cancer patient and was the first one completed sequenced [5]. Based on information from previous published studies, the phylogenetic tree constructed from HPV 16 types gives rise to branches reflecting the geographical origin of them and they are classified as European, African 1, African 2, and Asian-American [9,29–31]. Several publications conducted worldwide analyze the possible relation of HPV 16 genome variations with increased risk for cervical neoplasia [32–36].

Few studies are available on epidemiology of HPV in Greece [37–40] and this is the first to our knowledge on identification of molecular variants. According to the results of the present study, using MY09/11 sequence analysis of 104 HPV 16 samples, of the 9 variants analyzed, 6 new nucleotide variations were identified. Of the 12 nucleotide substitutions, 6 were not found in published studies while 3 of them resulted in non-synonymous nucleotide changes. According to studies in Europe [41,42] there is strong association of non-European isolates with the severity of the lesion and probably with oncogenesis. In this work, although the study population consisted of few African variants, the cytological examination mainly gave a HGSIL result. The results showed a high prevalence of the European lineage (Figure 1a). The European (HPV16 HM596520) variant is the most prevalent, as 84.6% of the samples of HPV 16, belong to that (Table 1) and associated with HGSIL lesions (*x*^2^ = 0.036, *P* < 0.05). Also in 6/6 cases of SCC and in 81.7% of LGSIL included in this study the European (HPV16 HM596520) variant was detected. HPV 31 is considered the fourth most common HPV type in squamous cervical carcinomas and in asymptomatic patients worldwide [2]. In the present study the molecular analysis of HPV 31 detected 11 nucleotide variations, 1 (A6647C) of them being presented for the first time. The phylogenetic analysis of *L1* (MY9/11) genomic part revealed that the HPV 31 variants detected to circulate in Greece are clustered into 3 different lineages according to Chen [7]. The new variant (A6647C) HM596536 was grouped with African and American variants. The most prevalent variant was of HM596540.

The molecular analysis of HPV 33 detected 4 nucleotide substitutions and 1 non-synonymous amino acid change. One of the four nucleotide substitutions a A-to-G at position 6673 was not previously published and was detected in isolate HM596529 making this variant most divergent from the others (Figure 1c).

Human papillomavirus type 18 (HPV 18) and HPV 45 account for approximately 20% of all cervix cancers [2]. Studies of HPV 18 variants have identified three distinct groups: European (E), Asian-American (AA) and African (Af) and is supposed that HPV 18 has co-evolved with the three major human phylogenetic branches: Caucasians, Asians and Africans [43]. Several publications suggest that HPV 18 variation can interfere with oncogenic potential and viral persistence [44–47] necessitating the analysis of HPV 18 variants worldwide. As far as HPV 45 is concerned little variability data are available although commonly found in cervical cancers.

In our report, only 5 samples with HPV 18 single infections were collected and 2 variants came up. All 7 nucleotide substitutions and the non-synonymous nucleotide change arose were previously published [11,23,48]. The most prevalent was HM596525 which phylogenetically was closer to non-African strains (Figure 1e). In the first clade that included variants of African origin, the isolate HM596526 was clustered with variants from Benin and Uganda that have been submitted to GenBank by Stewart *et al*. 1996 [11] and other African variants that have been submitted to GenBank by Chen *et al*. 2009 [6]. The other clade created, includes non-African variants, with European American and Asian American origin submitted to GenBank by the same laboratories. Based on the data of this report HPV 45 variants are distributed in two distinct lineages, A and B according to Chen *et al*. [6]. The HM596532 seems to vary from the rest ones and clusters in lineage B along with the HPV 45 reference strain. None of our variants were close to the phylogenetic sublineage consisting of African variants.

## 5. Conclusions

This is the first Greek study of nucleotide distribution and phylogenesis of HPV 16, 18, 31, 33 and 45, giving important information as 8 new *L1* single nucleotide changes were identified. Limitation of the present study should be taken under consideration, as the sample population of HPV 33, 18 and 45 was rather small due to the small number of single infections included in our HPV DNA bank. The phylogenetic analyses were done in an extensive way, using several different approaches. These analyses confirmed the observation that MY part of the HPV *L1* gene is informative enough to phylogenetically evaluate high-risk HPVs. Moreover, phylogenetic analyses of Greek isolates and isolates originating from other parts of the world confirmed the previous observations of geographical clustering of HPV 16 and HPV 18 isolates. Finally, it is considered to be of national importance that 84.6% of the samples of HPV16 belong to European variant (HM596520).

## Figures and Tables

**Table 1:** Distribution of HPV 16, 31, 33, 18, 45 variants according to Bethesda Cytological Diagnosis and the number of samples that belong to each variant.

	WNL	ASCUS	LGSIL	HGSIL	SCC	Total
**HPV 16 variants (sample)**	***n*= 11(10.6)**	***n*= 5 (4.8)**	***n*= 33 (31.7)**	***n*= 49 (47.1)**	***n*= 6 (5.8)**	***n*= 104**
E (HPV16 HM596508)				1 (2.0)		1 (0.96)
E (HPV16 HM596511)		1 (20.0)				1 (0.96)
E (HPV16 HM596513)		1 (20.0)				1 (0.96)
E (HPV16 HM596521)			3 (9.1)			3 (2.88)
E (HPV16 HM596522)			1 (3.0)	1 (2.0)		2 (1.92)
E (HPV16 HM596518)			1 (3.0)			1 (0.96)
E (HPV16 HM596520)	10 (90.1)	3 (60.0)	27 (81.7)	42 (85.7)	6 (100)	88 (84.6)
Non E (HPV16 HM596515)	1 (9.1)			1 (2.0)		2 (1.92)
Non E (HPV16 HM596512)			1 (3.0)	4 (8.1)		5(4.82)

**HPV 31 variants (sample)**	***n*= 5 (12.5)**	***n*= 6 (15)**	***n*= 23 (57.5)**	***n*= 5 (12.5)**		**40**
A (HPV31 HM596535)			1 (4.3)	1 (20.0)		2(5)
B (HPV31 HM596536)	1 (20)					1 (2.5)
B (HPV31 HM596539)	2 (40.0)	1 (16.7)	2 (8.6)	1 (20.0)		6 (15)
C (HPV31 HM596537)			1 (4,3)			1 (2.5)
C (HPV31 HM596538)			4 (17.4)			4 (10)
C (HPV31 HM596540)	2 (40.0)	5 (83.3)	15 (65.2)	3 (60.0)		26 (65)

**HPV 33 variants (sample)**	***n*= 1 (14.3)**	***n*= 1 (14.3)**	***n*= 2 (40.0)**	***n*= 3 (42.9)**		**7**
A (HPV33 HM596530)			1 (50.0)	3 (100.0)		4 (57.1)
B (HPV33 HM596527)		1 (100.0)				1 (14.3)
B (HPV33 HM596528)	1 (100.0)					1 (14.3)
C (HPV33 HM596529)			1 (50.0)			1 (14.3)

**HPV 18 variants (sample)**	***n*= 3 (60.0)**		***n*= 2 (40.0)**			**5**
Non Af (HPV18 HM596525)	2 (66.7)		2 (100.0)			4 (80.0)
Af (HPV18 HM596526)	1 (33.3)					1 (20.0)

**HPV 45 variants (sample)**	***n*= 1 (25.0)**		***n*= 2 (50.0)**		***n*= 1 (25.0)**	**4**
A (HPV45 HM596532)	1 (100.0)					1 (25.0)
B (HPV45 HM596531)			1 (50.0)			1 (25.0)
B (HPV45 HM596533)					1 (100.0)	1 (25.0)
B (HPV45 HM596534)			1 (50.0)			1 (25.0)

**Table 2:** Nucleotide and amino acid sequence variation in sequences of HPV 16 (**a**), 31 (**b**), 33 (**c**), 18 (**d**), 45 (**e**). The HPV16, 31, 33, 18, 45 *L1* variants were identified in 104, 40, 33, 5 and 4 samples from Greek women respectively. Nucleotide positions of detected changes are written vertically across the top and are indicated by the corresponding nucleotide letter. The numbering refers to the first nucleotide of each specific HPV reference sequence and the reference sequence used for each type is indicated as ref. Amino acid changes are signed above the nucleotide position. The number of isolates with identical sequences is listed in the first column.

Isolates with identicqal sequences (**a**)	**HPV16** (104 samples)	6592	6595	6694	6695	6721	6803	6824	6854	6865	6888	6914	6973
	HPV16 Ref (K02718)	C	C	A	A	G	A	T	C	C	C	A	C
	Non-synonymous mutations			E352D	T353P		T389S	S396P S396A			T417I		
1	HPV16 HM596508	T	T	.	.	.	.	C	.	.	.	.	.
1	HPV16 HM596511	T	T	.	.	.	.	.	.	.	.	G	.
5	HPV16 HM596512	T	T	.	C	A	.	.	T	T	.	.	T
1	HPV16 HM596513	T	T	.	.	.	.	G	.	.	.	.	.
3	HPV16 HM596521	T	T	.	.	.	.	.	.	.	T	.	NI
2	HPV16 HM596522	T	T	.	.	.	.	.	T	.	.	.	.
2	HPV16 HM596515	T	T	.	C	A	T	.	T	T	.	.	T
1	HPV16 HM596518	T	T	C	.	.	.	.	.	.	.	.	.
88	HPV16 HM596520	T	T	.	.	.	.	.	.	.	.	.	.

Isolates with identicqal sequences (**b**)	**HPV 31**(40 samples)	6511	6568	6586	6647	6664	6703	6772	6796	6817	6846	6862
	HPV31 Ref (J04353)	C	T	T	A	T	A	G	G	C	C	C
	Non-synonymous mutations										T432S	
2	HPV31 HM596535	T	.	.	.	.	.	.	.	A	G	.
1	HPV31 HM596536	T	C	.	C	.	.	A	A	A	.	T
1	HPV31 HM596537	T	.	G	.	.	G	.	A	A	.	.
4	HPV31 HM596538	T	.	G	.	C	.	.	A	A	.	.
6	HPV31 HM596539	T	C	.	.	.	.	A	A	A	.	T
26	HPV31 HM596540	T	.	G	.	.	.	.	A	A	.	.

Isolates with identicqal sequences (**c**)	**HPV33** (7 samples)	6664	6673	6748	6841
	HPV33 Ref (M12732)	A	A	A	C
	Non-synonymous mutations			E385D	
1	HPV33 HM596527	G	.	C	.
1	HPV33 HM596528	G	.	.	.
1	HPV33 HM596529	G	G	.	T
4	HPV33 HM596530	.	.	.	.

Isolates with identicqal sequences (**d**)	**HPV18** (5 samples)	6579	6581	6626	6719	6749	6842	6917
	HPV18 Ref (X05015)	G	T	C	G	G	C	G
	Non-synonymous mutations	V384I						
4	HPV18 HM596525	.	.	.	.	.	G	.
1	HPV18 HM596526	A	C	T	A	A	G	A

Isolates with identicqal sequences (**e**)	**HPV45** (4 samples)	6615	6665	6676	6687	6705	6816	6837	6861	6867
	HPV45 Ref (X74479)	A	A	A	C	G	A	C	G	T
	Non-synonymous mutations		N383T	S389G		Q392H				
1	HPV45 HM596531	.	.	G	T	C	G	A	A	C
1	HPV45 HM596532	.	.	G	.	.	G	.	A	.
1	HPV45 HM596533	G	.	G	T	C	G	A	A	.
1	HPV45 HM596534	G	C	G	T	C	G	A	A	.

NI: non informative.
